# Increased Blood Levels of NfL, GFAP, and Placental Growth Factor After Radiotherapy to the Brain

**DOI:** 10.1002/acn3.70278

**Published:** 2025-12-09

**Authors:** Erik Fernström, Thomas Björk‐Eriksson, Pontus Erickson, Jan Nyman, Henrik Zetterberg, Marie Kalm

**Affiliations:** ^1^ Department of Oncology Institute of Clinical Sciences, Sahlgrenska Academy at the University of Gothenburg Gothenburg Sweden; ^2^ Department of Oncology Sahlgrenska University Hospital Gothenburg Sweden; ^3^ Department of Psychiatry and Neurochemistry, Institute of Neuroscience and Physiology Sahlgrenska Academy at the University of Gothenburg Mölndal Sweden; ^4^ Clinical Neurochemistry Laboratory Sahlgrenska University Hospital Mölndal Sweden; ^5^ Department of Neurodegenerative Disease UCL Institute of Neurology, Queen Square London UK; ^6^ UK Dementia Research Institute at UCL London UK; ^7^ Hong Kong Center for Neurodegenerative Diseases, InnoHK Hong Kong China; ^8^ Wisconsin Alzheimer's Disease Research Center University of Wisconsin School of Medicine and Public Health, University of Wisconsin‐Madison Madison Wisconsin USA; ^9^ Department of Pharmacology Institute of Neuroscience and Physiology, Sahlgrenska Academy at the University of Gothenburg Gothenburg Sweden

**Keywords:** glial fibrillary acidic protein (GFAP), neurofilament light (NfL), placental growth factor (PlGF), prophylactic cranial irradiation (PCI), radiation‐induced CNS injury

## Abstract

In this study, we analyzed biomarkers of neuronal, glial, and vascular injury in longitudinal paired samples of blood and cerebrospinal fluid after prophylactic cranial irradiation in patients with small cell lung cancer. Neurofilament light chain protein (NfL) and glial fibrillary acidic protein (GFAP) increased in serum and cerebrospinal fluid after irradiation; serum NfL correlated with cerebrospinal fluid values, apparently independent of blood–brain barrier function, whereas GFAP correlations were weaker. Although several patients developed brain metastases, linear mixed model results were consistent with an independent effect of radiotherapy on serum NfL and GFAP. Serum placental growth factor also rose and correlated with the albumin ratio. Our results support a radiotherapy‐associated increase of NfL and GFAP in blood.

## 1 Introduction

Neurofilament light chain protein (NfL) and glial fibrillary acidic protein (GFAP) constitute major components of the neuronal and astrocytic cytoskeletons, respectively. NfL is crucial for axonal morphologic integrity and is increased in cerebrospinal fluid (CSF) following neuronal injury [1]. GFAP supports astrocytic structure and function and plays a key role in morphological alterations during astrocyte activation [2]. Both NfL and GFAP are established CSF biomarkers of brain pathology, and recent advances have enabled detection of these proteins at lower concentrations in blood [1, 3].

We have previously demonstrated increased NfL and GFAP levels in CSF after prophylactic cranial irradiation (PCI) in patients with small cell lung cancer (SCLC) [4], suggesting their potential utility as biomarkers of radiation‐induced brain injury. Since then, NfL and GFAP in blood have been studied in selected neuro‐oncological settings, including glioma [5] and brain metastases [6].

This study aimed to investigate the longitudinal trajectories of NfL and GFAP in blood after cranial radiotherapy using archival serum samples from the SCLC patients undergoing PCI. Additionally, paired CSF samples were re‐analyzed to explore correlations between blood and CSF concentrations. We also included the analysis of placental growth factor (PlGF), a candidate biomarker for neurovascular injury [7], in both blood and CSF samples.

## 2 Methods and Materials

The study was approved by the regional ethics review board (no. 194‐7), Gothenburg, Sweden, and by the Swedish Ethical Review Authority (no. 2023‐07422‐02). The samples were collected between 2010 and 2013. Patient characteristics and radiotherapy techniques have been described in detail previously [4, 8]. Eleven patients were included and had baseline assessment (8 female, 3 male; median age 67 years, inter‐quartile range (IQR) 64–74). Six patients had limited disease (LD) and five had extensive disease (ED). All had received carboplatin combined with either etoposide or irinotecan, and six had received thoracic radiotherapy.

Three patients were excluded from longitudinal analyses due to disease progression (*n* = 2) or ischemic stroke (*n* = 1). Consequently, eight patients had paired samples of CSF and serum at baseline as well as 3 months (median 86 days; IQR 78–99 days) after the end of radiotherapy. Additionally, five patients had CSF and four patients had serum samples available approximately 12 months post‐treatment.

In the eight patients with follow‐up assessments, PCI was administered on consecutive weekdays as 30 Gy in 15 fractions (LD, *n* = 5) or 20 Gy in 5 fractions (ED, *n* = 3), with concurrent thoracic radiotherapy (33 Gy in 11 fractions) in two patients. Subsequently, five of the eight patients developed brain metastases. One patient had a metastasis on the study‐specific baseline MRI. Two additional patients had small non‐specific contrast enhancements that later progressed to manifest metastases. Two other patients had brain metastasis at three and 12 months, respectively. One patient started second‐line chemotherapy (Hycamtin) prior to the three‐month follow‐up.

### 2.1 Analysis of Fluid Samples

CSF and serum samples were collected at the same visit in the neurology outpatient clinic at the Sahlgrenska University Hospital. Ten–12 mL of CSF was collected in polypropylene tubes, as described before [4], and serum was collected in serum separator gel tubes. Serum samples were centrifuged according to the manufacturer's instructions. CSF was analyzed for cell count and albumin, and serum was analyzed for albumin, and the samples were aliquoted and stored in the biobank at −80°C pending further analysis. The aliquots used for the present study had not been thawed before. CSF and serum samples were analyzed using the Simoa HD‐X instrument with the Neuro‐2‐Plex B kit (NfL & GFAP) and the Simoa PlGF Discovery kit (both from Quanterix, Billerica, MA). Previously reported albumin levels were used to calculate the CSF/serum albumin ratio (CSF albumin (mg/L)/serum albumin (g/L)). Samples were analyzed as singlicates. All measurements were performed in one round of experiments using one batch of reagents by board‐certified laboratory technicians who were blinded to clinical data. Intra‐assay coefficients of variation were below 10%.

### 2.2 Statistical Analysis

The Wilcoxon signed ranks test was used for the comparison of baseline and follow‐up samples. Visual inspection and the Shapiro–Wilk test indicated a non‐normal distribution of NfL, GFAP, and albumin ratio, but not of PlGF. Thus, NfL, GFAP, and albumin ratio values were transformed by their natural logarithm (Log_e_) for subsequent regression and correlation analyses. The effects of radiotherapy and brain metastases on the longitudinal changes in serum biomarker concentrations from baseline to 3 months were explored using linear mixed‐effects models, including all 11 patients. Time (coded as 0 or 1 for baseline and 3 months, respectively), brain metastasis status (coded as 0 for never or 1 for ever having a metastasis), and their interaction were included as fixed effects, and a random intercept for subject ID was included to account for repeated measures. The dependent variables were ln‐transformed NfL and ln‐transformed GFAP, as well as non‐transformed PlGF. Models were fitted with the “lme4” package (v 35.5) in R using restricted maximum likelihood with Satterthwaite's approximation for degrees of freedom. Estimated marginal means (EMMs) and within‐group contrasts were obtained using the “emmeans” package (v 1.11.2‐8), and results were back‐transformed to express geometric mean ratios (fold changes) on the original scale. Model assumptions were checked by visual inspection using the “performance” package (v 0.15.1). For correlation analysis of repeated measurements of paired samples, we employed the R implementation (“rmcorr” package v 0.7.0) [9] of the repeated measures correlation analysis as described in [10], including all eight patients with follow‐up samples. A 95% confidence interval (CI) is given for the correlation coefficients. Analyses were performed using R v. 4.1.1 (R Core Team 2017, Vienna, Austria) and SPSS v 29 (IBM Corp. Armonk, NY, USA). Statistical significance was defined as *p* < 0.05.

## 3 Results

**FIGURE 1:**
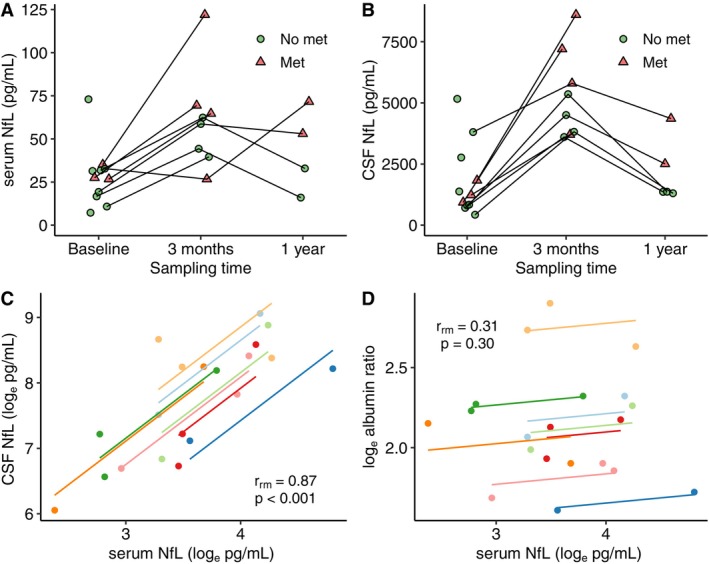
Longitudinal measurement of (A) serum NfL and (B) CSF NfL. Met = metastasis on MRI. Panels (C) and (D) show repeated measurements correlation of (C) serum NfL versus CSF NfL, (D) serum NfL versus albumin ratio.

**FIGURE 2:**
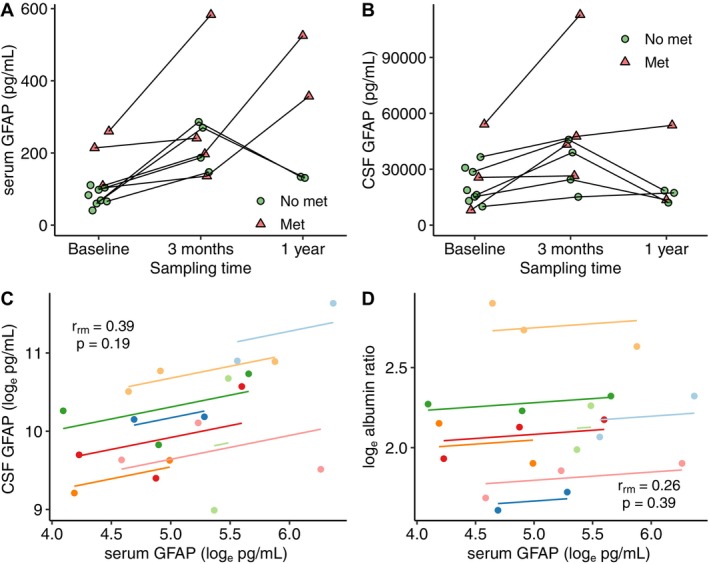
Longitudinal measurement of (A) serum GFAP and (B) CSF GFAP. Met = metastasis on MRI. Panels (C) and (D) show repeated measurements correlation of (C) serum GFAP versus CSF GFAP, (D) serum GFAP versus albumin ratio.

**FIGURE 3:**
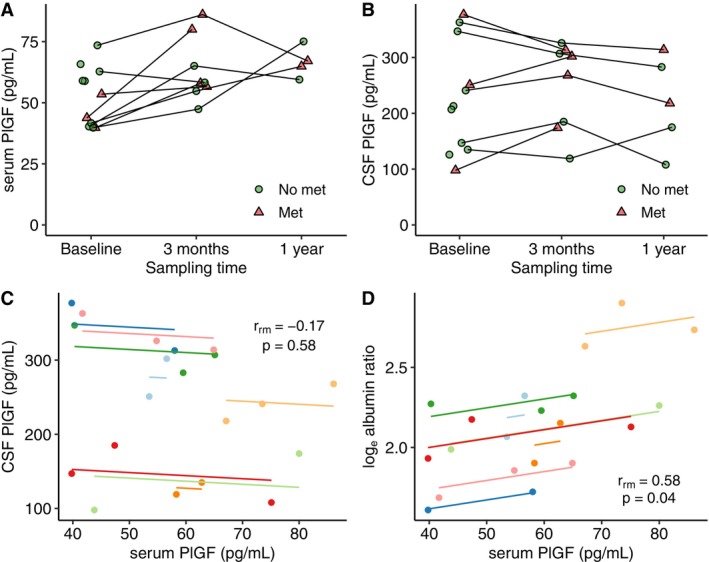
Longitudinal measurement of (A) serum PlGF and (B) CSF PlGF. Met = metastasis on MRI. Panels (C) and (D) show repeated measurements correlation of (C) serum PlGF versus CSF PlGF, (D) serum PlGF versus albumin ratio.

The longitudinal biomarker levels are shown in Table 1. NfL and GFAP levels significantly increased from baseline to the three‐month follow‐up (Figures 1 and 2 respectively), in both serum and CSF. PlGF was significantly increased in serum, but not in CSF, at 3 months (Figure 3).

**TABLE 1:** Biomarker values at baseline and follow‐up.

	Baseline (*n* = 11)	3 months (*n* = 8)	1 year (*n*[serum] = 4, *n*[csf] = 5)	Baseline versus 3 months
	Median (IQR)	Median (IQR)	Median (IQR)	Wilcoxon signed rank test
Serum NfL (pg/mL)	28 (17–33)	61 (41–68)	43 (20–67)	*p* = 0.017
CSF NfL (pg/mL)	1231 (807–2767)	4930 (3733–6849)	1368 (1334–3432)	*p* = 0.012
Serum GFAP (pg/mL)	98 (66–111)	219 (157–282)	246 (132–483)	*p* = 0.012
CSF GFAP (pg/mL)	18,790 (13,020–30,797)	41,129 (25,003–47,169)	17,336 (12,812–36,062)	*p* = 0.012
Serum PlGF (pg/mL)	54 (40–63)	58 (55–76)	66 (61–73)	*p* = 0.025
CSF PlGF (pg/mL)	213 (135–347)	285 (177–312)	218 (142–299)	*p* = 0.78

The full results of the linear mixed models can be found in File S1. For log‐NfL, there was a significant main effect of time (*β* = 0.91, 95% CI 0.14–1.68, *p* = 0.028), indicating higher levels at 3 months. The effects of brain metastases and the time × metastases interaction were not significant. Back‐transformed estimated marginal means (EMMs) showed a 2.49‐fold increase (95% CI 1.19–5.20, *p* = 0.022) in patients without metastases and a 2.21‐fold increase (95% CI 1.21–4.02, *p* = 0.018) in those with metastases.

For log‐GFAP, the main effects of both time (*β* = 1.23, 95% CI 0.80–1.66, *p* < 0.001) and metastasis status (*β* = 0.74, 95% CI 0.16–1.33, *p* = 0.017) were significant, and the interaction was negative and significant (*β* = −0.74, 95% CI −1.30 to 0.19, *p* = 0.016), indicating a smaller increase in patients with metastases. EMMs showed a 3.42‐fold increase (95% CI 2.20–5.32, *p* < 0.001) in patients without metastases and a 1.63‐fold increase (95% CI 1.14–2.32, *p* = 0.015) among those with metastases.

For PlGF, which was analyzed on the original scale, the linear mixed‐effects model revealed no significant main effects of time, metastasis status, or their interaction (all *p* > 0.05).

In a repeated measures correlation analysis, NfL in serum and CSF showed a common intra‐individual association (*r*_rm_ = 0.87, CI 0.60–0.96, *p* < 0.001, Figure 1C). Analysis of serum NfL versus CSF/serum albumin ratio did not show a significant correlation (*r*_rm_ = 0.31, CI −0.29 to 0.73, *p* = 0.30, Figure 1D).

Correlation analyses of GFAP in serum and CSF (*r*_rm_ = 0.39, CI −0.21 to 0.77, *p* = 0.19) as well as between serum GFAP and albumin ratio (*r*_rm_ = 0.26, CI −0.34 to 0.71, *p* = 0.39) are shown in Figure 2C,D. The results were consistent with a moderate but non‐significant correlation between serum and CSF GFAP.

There was no correlation between serum and CSF levels of PlGF (*r*_rm_ = −0.17, CI −0.66 to 0.42, *p* = 0.58, Figure 3C). However, serum PlGF showed a significant correlation with the CSF/serum albumin ratio (*r*_rm_ = 0.58, CI 0.05–0.86, *p* = 0.04, Figure 3D).

Using log‐transformed values, both NfL (*r*_rm_ = 0.99, CI 0.95–1, *p* < 0.01) and GFAP (*r*_rm_ = 0.73, CI 0.30–0.91, *p* < 0.01) demonstrated significant correlations between the results obtained in the present analyses and the previously reported CSF levels obtained in the same samples when analyzed in 2013 [4].

## 4 Discussion

This study has demonstrated significant increases in serum NfL and GFAP following PCI in SCLC patients, consistent with previous CSF‐based findings [4]. Exploratory linear mixed model results suggested an independent treatment‐related effect. A significant correlation between serum and CSF NfL levels was found, which seemed independent of blood–brain barrier (BBB) function. Prior studies support this finding, indicating that blood NfL levels correlate well with CSF concentrations across various neurological conditions, regardless of BBB integrity [11, 12, 13]. PlGF has been shown to be a potential biomarker of vascular contributions to dementia [7]. The contribution of PlGF to cognitive decline in these conditions seems to be partially mediated through BBB disruption [14]. We observed a moderate increase of PlGF in blood and a correlation with CSF/serum albumin ratio. However, the linear mixed model did not support a significant treatment effect. Interpretation of these findings is complicated by the potential release of PlGF from non‐CNS sources, including systemic tumor burden and brain metastases [15, 16]. Thus, the value of PlGF as a biomarker of radiation‐induced brain injury requires further investigation in larger studies.

The study's limited sample size and potential confounders, including brain metastases, should be acknowledged. The numerically higher biomarker levels in patients who developed brain metastases are consistent with recent findings from other groups [6, 17]. Additionally, prior chemotherapy could have influenced baseline biomarker levels [18], however, the observed increase post‐PCI is unlikely to have been significantly impacted by previous treatments, and only one patient received systemic therapy between baseline and the 3‐month follow‐up. The contribution from the peripheral nervous system to the serum levels of NfL and GFAP during PCI itself is probably limited since the radiation dose to the CNS far outweighs the doses to peripheral nerves in this situation. However, concurrent thoracic radiotherapy in two patients may have introduced additional variability. Although there was no systematic follow‐up of renal function, the longitudinal design somewhat ameliorates this potential bias. Available data suggest an acceptable stability of both NfL and GFAP in blood samples during long‐term storage [19, 20]. NfL in CSF seems to be relatively resistant to both long‐term storage [21] and freeze–thaw cycles [22]. However, CSF GFAP has been shown to be quite sensitive to pre‐analytical variations in handling [22, 23]. Taken together, this is consistent with the good correlation we observed between CSF NfL levels analyzed for the present report and our previously reported CSF NfL levels analyzed in 2013 in the same patients [4]. Sensitivity of GFAP in CSF to long‐term storage could have contributed to the relatively moderate correlation observed for CSF GFAP between our present and previous results, as well as the weak correlation between GFAP in blood and CSF in the present study. It is unclear to what extent the levels of PlGF may have been influenced by protein degradation during storage.

In conclusion, our findings are consistent with an independent effect of cranial radiotherapy on serum levels of NfL and GFAP. Further studies are needed to explore dose–response relationships and correlations with clinical toxicity.

## Author Contributions

**Erik Fernström:** investigation, methodology, formal analysis, writing – original draft. **Thomas Björk‐Eriksson:** conceptualization, funding acquisition, project administration, supervision, writing – review and editing. **Pontus Erickson:** methodology, validation, writing – review and editing. **Jan Nyman:** conceptualization, investigation, supervision, writing – review and editing. **Henrik Zetterberg:** conceptualization, funding acquisition, methodology, writing – review and editing. **Marie Kalm:** project administration, resources, supervision, funding acquisition, writing – review and editing.

## Funding

This work was supported by AD Strategic Fund and the Alzheimer's Association (ADSF‐21‐831376‐C, ADSF‐21‐831377‐C, ADSF‐21‐831381‐C, ADSF‐24‐1284328‐C), Alzheimer's Drug Discovery Foundation (201809–2016862), HORIZON EUROPE Research and Innovation program (101053962), Vetenskapsrådet (2019‐02397, 2022‐01018, 2023‐00356), Lions Cancer Fund of Western Sweden, Frimurare Barnhus Foundations of Gothenburg, Stiftelsen Jubileumsklinikens Forskningsfond mot Cancer, Barncancerfonden, Svenska Läkaresällskapet, Swedish state under the ALF‐agreement (ALFGBG‐71320, ALFGBG‐774991, ALFGBG‐819811, ALFGBG‐875691), European Partnership on Metrology, European Union's Horizon Europe Research and Innovation Program and by the Participating States (NEuroBioStand, #22HLT07), Cure Alzheimer's Fund, the Bluefield Project, the Olav Thon Foundation, the Erling‐Persson Family Foundation, Familjen Rönströms Stiftelse, Stiftelsen för Gamla Tjänarinnor, Hjärnfonden, Sweden, the European Union's Horizon 2020 research and innovation program under the Marie Skłodowska‐Curie grant agreement No 860197 (MIRIADE), the European Union Joint Program—Neurodegenerative Disease Research (JPND2021‐00694), the National Institute for Health and Care Research University College London Hospitals Biomedical Research Centre, the UK Dementia Research Institute at UCL (UKDRI‐1003), and an anonymous donor.

## Conflicts of Interest

Henrik Zetterberg has served on scientific advisory boards and/or as a consultant for Abbvie, Acumen, Alector, Alzinova, ALZpath, Amylyx, Annexon, Apellis, Artery Therapeutics, AZTherapies, Cognito Therapeutics, CogRx, Denali, Eisai, Enigma, LabCorp, Merry Life, Nervgen, Novo Nordisk, Optoceutics, Passage Bio, Pinteon Therapeutics, Prothena, Quanterix, Red Abbey Labs, reMYND, Roche, Samumed, Siemens Healthineers, Triplet Therapeutics, and Wave, has given lectures sponsored by Alzecure, BioArctic, Biogen, Cellectricon, Fujirebio, Lilly, Novo Nordisk, Roche, and WebMD, and is a co‐founder of Brain Biomarker Solutions in Gothenburg AB (BBS), which is a part of the GU Ventures Incubator Program (outside submitted work). Marie Kalm is an employee at AstraZeneca. The remaining authors have no conflicts of interest to disclose.

## Supporting information


**File S1:** Results of linear mixed models of serum biomarkers.

## Data Availability

The data that support the findings of this study are available on request from the corresponding author. The data are not publicly available due to privacy or ethical restrictions.
