# Supplementary material for: Increased Blood Levels of NfL, GFAP, and Placental Growth Factor After Radiotherapy to the Brain
Source: Ann Clin Transl Neurol. 2025 Dec 9;13(2):399–404. doi: 10.1002/acn3.70278 (PMC12883678; doi:10.1002/acn3.70278)
Supplement: Supplementary file 1 — File S1: Results of linear mixed models of serum biomarkers. [file ACN3-13-399-s001.pdf]

Supplementary file 1: Linear mixed models of serum biomarkers

serum NfL

*Model: log-NfL ~ Timepoint * Metastasis status + (1|id); Estimation: REML; Random intercept for id.*
Fit: AIC = 43.4, Marginal R^2 = 0.359, Conditional R^2 = 0.745

Random effects			
**Groups**	**Name**	**Variance**	**SD**
id	(Intercept)	0.228	0.477
Residual		0.151	0.388

Estimates of fixed effects						
**Fixed effect**	**Estimate (β)**	**95% CI**	**SE**	**df**	**t value**	**p value**
(Intercept)	3.061	[2.501, 3.622]	0.251	9.942	12.185	0.000
Timepoint	0.913	[0.143, 1.683]	0.301	5.078	3.032	0.028
Metastasis status	0.260	[-0.571, 1.091]	0.373	9.942	0.698	0.501
Timepoint:metastasis status	-0.120	[-1.145, 0.905]	0.388	4.604	-0.309	0.771

Pairwise contrasts; timepoint 1 - timepoint 0, within each metastasis status group						
**Metastasis status**	**Estimate (Δ on model scale)**	**95% CI (model scale)**	**t ratio**	**p value**	**Ratio (back-transformed)**	**95% CI (back-transformed)**
0	0.913	[0.178, 1.648]	2.907	0.022	2.491	[1.194, 5.195]
1	0.793	[0.194, 1.392]	3.226	0.018	2.209	[1.214, 4.021]

serum GFAP

*Model: log-GFAP ~ Timepoint * Metastasis status + (1|id); Estimation: REML; Random intercept for id.*
Fit: AIC = 31.4, Marginal R^2 = 0.602, Conditional R^2 = 0.890

Random effects			
**Groups**	**Name**	**Variance**	**SD**
id	(Intercept)	0.138	0.372
Residual		0.053	0.230

Estimates of fixed effects						
**Fixed effect**	**Estimate (β)**	**95% CI**	**SE**	**df**	**t value**	**p value**
(Intercept)	4.225	[3.832, 4.618]	0.178	10.899	23.686	0.000
Timepoint	1.230	[0.801, 1.658]	0.181	6.944	6.799	0.000
Metastasis status	0.744	[0.161, 1.327]	0.265	10.899	2.814	0.017
Timepoint:metastasis status	-0.743	[-1.299, -0.188]	0.232	6.587	-3.205	0.016

Pairwise contrasts; timepoint 1 - timepoint 0, within each metastasis status group						
**Metastasis status**	**Estimate (Δ on model scale)**	**95% CI (model scale)**	**t ratio**	**p value**	**Ratio (back-transformed)**	**95% CI (back-transformed)**
0	1.230	[0.787, 1.672]	6.584	0.000	3.420	[2.196, 5.324]
1	0.486	[0.132, 0.841]	3.350	0.015	1.626	[1.141, 2.319]

serum PlGF

*Model: PlGF ~ Timepoint * Metastasis status + (1|id); Estimation: REML; Random intercept for id.*
Fit: AIC = 135.3, Marginal R^2 = 0.214, Conditional R^2 = 0.599

Random effects			
**Groups**	**Name**	**Variance**	**SD**
id	(Intercept)	79.07	8.892
Residual		82.12	9.062

Estimates of fixed effects						
**Fixed effect**	**Estimate (β)**	**95% CI**	**SE**	**df**	**t value**	**p value**
(Intercept)	54.433	[43.227, 65.639]	5.183	12.904	10.502	0.000
Timepoint	5.836	[-10.018, 21.690]	6.912	8.252	0.844	0.422
Metastasis status	-3.973	[-20.594, 12.648]	7.688	12.904	-0.517	0.614
Timepoint:metastasis status	10.804	[-10.125, 31.734]	8.979	7.536	1.203	0.265

Pairwise contrasts; timepoint 1 - timepoint 0, within each metastasis status group				
**Metastasis status**	**Estimate (Δ on model scale)**	**95% CI (model scale)**	**t ratio**	**p value**
0	5.836	[-10.982, 22.654]	0.804	0.445
1	16.640	[2.700, 30.580]	2.903	0.026
